# Preoperative nutritional status, frailty, and postoperative delirium in patients undergoing pancreatic cancer surgery: a prospective observational study

**DOI:** 10.3389/fnut.2026.1825296

**Published:** 2026-06-19

**Authors:** Ningning Xia, Lulu Ding, Neng Shi, Yuan Song, Kuei-Ching Pan

**Affiliations:** 1 Pancreas Center, The Affiliated BenQ Hospital of Nanjing Medical University, Nanjing, Jiangsu, China; 2 School of Nursing, Nanjing Medical University, Nanjing, Jiangsu, China

**Keywords:** frailty, mediation analysis, nutrition, pancreatic cancer, postoperative delirium, prognostic nutritional index

## Abstract

**Background:**

Postoperative delirium (POD) is a common neuropsychiatric complication after major abdominal surgery and is associated with adverse clinical outcomes. Patients undergoing pancreatic cancer surgery are particularly vulnerable to nutritional impairment and physiological decline, both of which may increase susceptibility to POD. Frailty reflects reduced physiological reserve and may be associated with both poor nutritional status and postoperative cognitive vulnerability. However, whether frailty helps explain the association between preoperative nutritional status and POD remains unclear. This study aimed to investigate the association between preoperative nutritional status and POD and to explore the role of frailty in this relationship.

**Methods:**

A prospective observational study was conducted among patients who underwent elective pancreatic cancer surgery at a tertiary hospital in Jiangsu Province, China, between August 2023 and March 2024. Preoperative nutritional status was assessed using the prognostic nutritional index (PNI), and frailty was evaluated using the FRAIL scale. POD was assessed twice daily from postoperative day 1 to postoperative day 4 using the Confusion Assessment Method (CAM). Multivariable logistic regression was performed to identify factors independently associated with POD. Mediation analysis was conducted using PROCESS macro version 5.0 (Model 4) with 5,000 bootstrap resamples to evaluate the potential mediating role of frailty in the association between PNI and POD. Receiver operating characteristic (ROC) curve analysis was carried out to assess the predictive performance of PNI, frailty score, and their combined model.

**Results:**

A total of 293 patients were included, among whom 57 (19.5%) developed POD. In the multivariable logistic regression model, higher frailty score (OR = 2.451, 95% CI: 1.442–4.164, *p* = 0.001), older age (OR = 1.108, 95% CI: 1.038–1.182, *p* = 0.002), history of cerebral infarction (OR = 3.774, 95% CI: 1.003–14.195, *p* = 0.049), longer preoperative fasting time (OR = 2.440, 95% CI: 1.693–3.518, *p* < 0.001), and intraoperative hypotension (OR = 2.868, 95% CI: 1.155–7.122, *p* = 0.023) were independently associated with POD. PNI was significantly associated with frailty score (B = −0.0476, SE = 0.0097, *p* < 0.001). Mediation analysis showed a significant indirect effect of PNI on POD through frailty (effect = −0.0430, 95% bootstrap CI: −0.0866 to −0.0187), whereas the direct effect was not significant (B = −0.0039, SE = 0.0439, *p* = 0.9295), indicating an indirect-only mediation pattern. ROC analysis showed that the area under the curve (AUC) was 0.661 for PNI, 0.829 for frailty score, and 0.835 for the combined model. The AUC of frailty score was significantly higher than that of PNI (DeLong test, *p* < 0.001), whereas the combined model did not significantly improve discrimination compared with frailty score alone (*p* = 0.473).

**Conclusion:**

Frailty was independently associated with postoperative delirium in patients undergoing pancreatic cancer surgery and serves as a potential pathway linking poor preoperative nutritional status to POD. Poorer nutritional status may be associated with POD susceptibility partly through frailty. Although adding PNI to frailty slightly increased the AUC, it did not provide statistically significant incremental discriminative value beyond frailty alone.

## 1 Introduction

Pancreatic cancer (PC) is one of the most lethal malignancies worldwide, with its incidence and mortality rates continuing to rise in recent years. It is projected that by 2030, pancreatic cancer will become the second leading cause of cancer-related death in the United States (1). In China, pancreatic cancer accounted for an estimated 118,700 new cases and 106,300 deaths in 2022 (2). Due to insidious onset and rapid progression, surgical resection remains the only potentially curative treatment capable of achieving long-term survival. However, pancreatic surgery is technically complex and highly invasive, and related to a relatively high incidence of postoperative complications (3). In addition to common physical complications such as pancreatic fistula, postoperative hemorrhage, delayed gastric emptying, and cardiopulmonary complications, neuropsychiatric complications have gradually attracted increasing clinical attention (4). Among these, postoperative delirium (POD) is a common acute neurocognitive disorder characterized by disturbances in attention, awareness, and cognition. The incidence of POD following pancreatic cancer surgery ranges from 14.0 to 30.5% (5, 6). Previous studies have shown that POD is linked to a range of adverse outcomes, including increased postoperative complications, prolonged hospital stay (7), increased healthcare costs (8), and higher mortality risk (9). In addition, persistent delirium may lead to long-term cognitive decline and a significant reduction in quality of life (10, 11).

In recent years, increasing attention has been directed toward potential risk factors for POD, including advanced age, higher American Society of Anesthesiologists (ASA) physical status classification, comorbidities, and perioperative physiological stress (12). However, beyond these traditional clinical factors, the overall physiological reserve of patients has also been recognized as an important determinant of postoperative vulnerability. Frailty is a multidimensional syndrome characterized by reduced physiological reserve and impaired resistance to stressors, and it has been extensively studied in surgical populations. Previous research has demonstrated that frailty is closely associated with adverse postoperative outcomes, including complications, mortality, and cognitive impairment, and has been identified as a major risk factor for postoperative delirium (5, 13–15).

Malnutrition is highly prevalent among patients with pancreatic cancer and is considered one of the most common nutritional disorders among patients with malignancies (16). Pancreatic tumors and pancreatic dysfunction frequently lead to impaired digestion and absorption, as well as metabolic disturbances, thereby increasing the risk of malnutrition (17). Nutritional status plays an important role in maintaining immune function and regulating inflammatory responses. In addition, emerging evidence suggests that nutrition-related factors may influence brain function through pathways such as systemic inflammation and the microbiota–gut–brain axis (18–20). Alterations in gut microbiota composition and intestinal function are also suggested to contribute to the development of frailty (21, 22).

The prognostic nutritional index (PNI), calculated based on serum albumin levels and peripheral lymphocyte counts, has been widely used as a convenient indicator to evaluate the immunonutritional status and prognosis of patients with cancer (23). Given the close relationships among nutritional status, frailty, and postoperative outcomes, preoperative nutritional status may be associated with postoperative delirium, and frailty may be involved in this association. However, current evidence on the relationships among nutritional status, frailty, and postoperative delirium remains limited. In particular, whether frailty helps explain the association between nutritional status and POD has not been systematically investigated in patients undergoing pancreatic surgery.

Therefore, this study aimed to explore the association between preoperative nutritional status and postoperative delirium in patients undergoing pancreatic cancer surgery and to further assess the role of frailty in this relationship. A better understanding of these associations may help identify high-risk patients and inform perioperative nutritional management and delirium prevention strategies.

## 2 Methods

### 2.1 Study design and participants

This prospective observational study was conducted at the Pancreatic Center of a tertiary Grade A hospital in Jiangsu Province, China. Consecutive patients who underwent elective pancreatic cancer surgery between August 2023 and March 2024 were screened for eligibility. Patients were eligible if they were aged 18 years or older, had provided written informed consent, underwent surgery under general anesthesia or combined epidural anesthesia, had an American Society of Anesthesiologists (ASA) physical status classification of I–III, were diagnosed with pancreatic malignancy confirmed by imaging or pathological examination, and underwent elective open total or partial pancreatectomy. Patients were excluded if they had a history of psychiatric or psychological disorders before surgery, had visual, auditory, or language impairments that prevented cooperation with study procedures, had preexisting dementia or Parkinson’s syndrome, or underwent other surgical procedures simultaneously. The study was approved by the Ethics Committee of BenQ Medical Center Affiliated to Nanjing Medical University (approval number: 2024-KL005).

### 2.2 Sample size calculation

Sample size was estimated using a single-proportion formula based on the expected incidence of postoperative delirium after pancreaticoduodenectomy (approximately 25%) (24). With a two-sided *α* of 0.05 and an allowable error of 0.05, the minimum required sample size was estimated to be 289 patients. A total of 293 patients were ultimately included. This estimation was used to guide recruitment based on the expected POD incidence rather than as a formal power calculation for multivariable regression or mediation analysis.

### 2.3 Ethical approval

This study was approved by the Ethics Committee of BenQ Medical Center Affiliated to Nanjing Medical University (approval number: 2024-KL005). Written informed consent was obtained from all participants or their legal representatives.

### 2.4 Data collection

A structured case report form was developed based on a literature review, group discussion, and consultation with pancreatic surgeons. Data were obtained from patient interviews, medical records, anesthesia records, and laboratory reports.

Preoperative data were collected on the day before surgery or supplemented from the most recent available preoperative medical records and laboratory test results. These variables included age, sex, education level, body mass index (BMI), current smoking, current alcohol use, hypertension, diabetes mellitus, history of previous surgery, history of cerebral infarction, preoperative pain, American Society of Anesthesiologists (ASA) physical status classification, nutritional risk, prognostic nutritional index (PNI), preoperative serum albumin, lymphocyte count, C-reactive protein, glycated hemoglobin (HbA1c), preoperative urea, preoperative creatinine, preoperative indirect bilirubin, preoperative total bilirubin, preoperative alanine aminotransferase (ALT), preoperative aspartate aminotransferase (AST), and frailty score. Intraoperative data were extracted from anesthesia records and included preoperative fasting time, intraoperative hypotension, blood loss, blood transfusion, surgical duration, and anesthesia duration. Postoperative data included postoperative direct bilirubin and postoperative delirium.

Nutritional risk was assessed using the Nutrition Risk Screening 2002 (NRS 2002), with a total score of 3 or higher indicating nutritional risk. PNI was calculated as serum albumin (g/L) + 5 × lymphocyte count (10^9/L). Preoperative frailty was assessed one day before surgery using the Frailty Screening Scale (FRAIL), which consists of five items evaluating fatigue, resistance, ambulation, illnesses, and loss of weight (25). Each item was scored as 0 (no) or 1 (yes), with a total score ranging from 0 to 5; higher scores indicate greater frailty. ASA physical status classification (I–III) was recorded preoperatively (26).

Postoperative delirium was assessed at the bedside twice daily from postoperative day 1 to postoperative day 4 by trained researchers using the Confusion Assessment Method (CAM). According to Shenkin et al. (27), patients were evaluated across four domains: mental status changes, attention, disorganized thinking, and level of consciousness. Delirium was diagnosed when acute onset or fluctuating course and inattention were present together with either disorganized thinking or altered level of consciousness. When the diagnostic criteria for delirium were met at least once, the diagnosis was confirmed after discussion with the attending physician, and the time of onset was recorded. Patients were classified as having POD if they met the diagnostic criteria at least once during the assessment period. Considering the fluctuating nature of delirium, and based on recommendations that POD screening should continue for at least the first 3 postoperative days and be performed at least twice daily, with evidence suggesting that extending surveillance to 4 days may further improve case detection, the surveillance window in this study was set from postoperative day 1 to postoperative day 4 (28).

Before formal data collection, two graduate students received standardized training and passed an assessment to ensure consistency in data collection and outcome evaluation. Data quality was checked regularly during the study period, and all data were double-checked by two researchers before entry.

### 2.5 Statistical analysis

Statistical analyses were performed using SPSS version 27.0. Descriptive statistics were first used to summarize the data. Continuous variables with a normal distribution were expressed as mean ± standard deviation (SD), and between-group comparisons were performed using the independent-samples t-test. Continuous variables with a non-normal distribution were expressed as median and interquartile range [M (P25, P75)] and were compared using the Mann–Whitney U test. Categorical variables were expressed as frequencies and percentages and were compared using the chi-square test or Fisher’s exact test, as appropriate.

To identify variables associated with postoperative delirium (POD), univariate logistic regression analyses were first performed. Variables with statistical significance in the univariate analyses (*p* < 0.05), together with prognostic nutritional index (PNI) and frailty score, were entered into a multivariable logistic regression model to identify independent factors associated with POD. Odds ratios (ORs) with 95% confidence intervals (CIs) were reported. Model calibration was assessed using the Hosmer-Lemeshow goodness-of-fit test.

To examine the association between preoperative nutritional status and frailty, a multivariable linear regression model was constructed with frailty score as the dependent variable. PNI was entered as the main independent variable, and age, primary education or less, history of hypertension, history of cerebral infarction, ASA III status, and preoperative fasting time were included as adjustment variables. Before multivariable regression analysis, multicollinearity among candidate variables was assessed using tolerance and variance inflation factor (VIF). A VIF value < 5 was considered to indicate no substantial multicollinearity. Mediation analysis was performed using PROCESS macro version 5.0 (Model 4) in SPSS. PNI was specified as the independent variable, frailty score as the mediator, and POD as the binary outcome. Age, hypertension, history of cerebral infarction, primary education or less, ASA III status, and preoperative fasting time were included as covariates. Total, direct, and indirect effects were estimated, and the indirect effect was assessed using 5,000 bootstrap resamples with 95% confidence intervals. Because POD was a binary outcome, effects on Y were expressed on a log-odds metric. As a sensitivity analysis, preoperative C-reactive protein was additionally included in the mediation model to assess the robustness of the indirect effect.

Receiver operating characteristic (ROC) curve analysis was conducted to evaluate the predictive performance of PNI, frailty score, and their combined model for POD. The combined model score was derived from the predicted probability of a logistic regression model including PNI and frailty score. The area under the curve (AUC), sensitivity, specificity, and optimal cut-off values were calculated, and the optimal cut-off value was determined using the Youden index. Pairwise comparisons of AUCs were performed using the DeLong test in R (version 4.5.3) with the pROC package, and the final ROC figure was generated in R. All statistical tests were two-sided, and *p* < 0.05 was considered statistically significant.

## 3 Results

### 3.1 Perioperative and clinical characteristics of patients with and without postoperative delirium

A total of 293 patients who underwent pancreatic surgery were included. Among them, 57 patients developed postoperative delirium and 236 did not, corresponding to an incidence of 19.5%. Univariate comparisons showed that the POD and non-POD groups differed significantly in age, educational level, history of hypertension, history of cerebral infarction, ASA physical status classification, nutritional risk, PNI, preoperative fasting time, intraoperative hypotension, and frailty-related indicators (all *p* < 0.05). Compared with the non-POD group, patients in the POD group were older, had lower educational level, higher prevalence of hypertension and cerebral infarction, higher ASA classification, lower PNI, and longer preoperative fasting time. Besides, they were more likely to experience intraoperative hypotension, and had more severe frailty (Table 1).

**Table 1:** Perioperative and clinical characteristics of patients with and without postoperative delirium.

Variable	Category	Overall (*n* = 293)	POD (*n* = 57)	Non-POD (*n* = 236)	Test statistic	*p* value
Age (years)		62.00 (56.00, 70.00)	75.00 (70.00, 77.00)	60.00 (54.00, 66.00)	−8.413	<0.001
Sex, *n* (%)	Male	170 (58.0)	28 (49.1)	142 (60.2)	1.869	0.172
	Female	123 (42.0)	29 (50.9)	94 (39.8)		
Education level	Primary or less	117 (39.9)	38 (66.7)	79 (33.5)	23.976	<0.001
	Middle school	94 (32.1)	13 (22.8)	81 (34.3)		
	High school	51 (17.4)	6 (10.5)	45 (19.1)		
	College or above	31 (10.6)	0 (0.0)	31 (13.1)		
BMI (kg/m^2^)	Underweight	23 (7.8)	7 (12.3)	16 (6.8)	4.700	0.195
	Normal	162 (55.3)	35 (61.4)	127 (53.8)		
	Overweight	87 (29.7)	13 (22.8)	74 (31.4)		
	Obese	21 (7.2)	2 (3.5)	19 (8.1)		
Current smoking	No	226 (77.1)	46 (80.7)	180 (76.3)	0.291	0.590
	Yes	67 (22.9)	11 (19.3)	56 (23.7)		
Current alcohol	No	243 (82.9)	45 (78.9)	198 (83.9)	0.484	0.487
	Yes	50 (17.1)	12 (21.1)	38 (16.1)		
Hypertension	No	178 (60.8)	25 (43.9)	153 (64.8)	7.611	0.006
	Yes	115 (39.2)	32 (56.1)	83 (35.2)		
Diabetes mellitus	No	218 (74.4)	40 (70.2)	178 (75.4)	0.417	0.518
	Yes	75 (25.6)	17 (29.8)	58 (24.6)		
History of previous surgery	No	157 (53.6)	33 (57.9)	124 (52.5)	0.336	0.562
	Yes	136 (46.4)	24 (42.1)	112 (47.5)		
History of cerebral infarction	No	273 (93.2)	45 (78.9)	228 (96.6)	19.829	<0.001
	Yes	20 (6.8)	12 (21.1)	8 (3.4)		
Preoperative pain	No	148 (50.5)	27 (47.4)	121 (51.3)	0.145	0.703
	Yes	145 (49.5)	30 (52.6)	115 (48.7)		
ASA physical status	I	3 (1.0)	0 (0.0)	3 (1.3)	38.345	<0.001
	II	207 (70.6)	22 (38.6)	185 (78.4)		
	III	83 (28.3)	35 (61.4)	48 (20.3)		
Nutritional risk (NRS)	No	191 (65.2)	21 (36.8)	170 (72.0)	23.529	<0.001
	Yes	102 (34.8)	36 (63.2)	66 (28.0)		
Prognostic nutritional index (PNI)		46.71 ± 5.53	44.38 ± 5.56	47.27 ± 5.38	3.619	<0.001
Preoperative serum albumin, g/L		39.80 (37.20, 42.00)	38.40 (35.80, 40.90)	40.15 (37.68, 42.32)	−3.161	0.002
Lymphocyte count, 10^9^/L		1.34 (1.00, 1.80)	1.14 (0.85, 1.49)	1.39 (1.06, 1.86)	−2.922	0.003
C-reactive protein, mg/L		3.20 (0.80, 9.70)	4.80 (1.30, 10.60)	3.05 (0.80, 9.18)	−1.364	0.173
HbA1c, %		6.00 (5.60, 6.80)	6.30 (5.50, 7.40)	5.90 (5.60, 6.70)	−0.752	0.452
Preoperative urea, mmol/L		5.18 (4.05, 6.55)	5.20 (3.94, 6.99)	5.17 (4.12, 6.52)	−0.053	0.958
Preoperative creatinine, μmol/L		64.00 (54.00, 79.00)	61.00 (50.00, 81.00)	65.00 (55.00, 78.25)	0.671	0.502
Preoperative indirect bilirubin, μmol/L		5.45 (3.83, 8.21)	5.86 (4.37, 9.27)	5.39 (3.73, 8.13)	−1.121	0.262
Preoperative total bilirubin, μmol/L		14.40 (9.60, 26.60)	16.70 (9.90, 48.70)	14.20 (9.57, 21.90)	−1.150	0.250
Postoperative direct bilirubin, μmol/L		6.69 (4.42, 16.58)	7.00 (5.20, 37.96)	6.50 (4.35, 12.40)	−1.934	0.053
Preoperative ALT, U/L		22.2 (13.3, 63.1)	27.2 (11.9, 127.0)	21.9 (13.8, 48.3)	−0.908	0.364
Preoperative AST, U/L		20.0 (15.1, 49.7)	23.5 (16.4, 102.9)	19.7 (14.8, 35.2)	−2.454	0.014
Preoperative fasting time, h		9.00 (8.50, 9.50)	10.00 (9.00, 12.00)	9.00 (8.50, 9.50)	−5.526	<0.001
Intraoperative hypotension, *n* (%)	No	232 (79.2)	34 (59.6)	198 (83.9)	16.377	<0.001
	Yes	61 (20.8)	23 (40.4)	38 (16.1)		
Blood loss, mL		150.00 (100.00, 200.00)	200.00 (100.00, 300.00)	150.00 (100.00, 200.00)	−1.366	0.172
Blood transfusion, *n* (%)	No	261 (89.1)	48 (84.2)	213 (90.3)	1.724	0.189
	Yes	32 (10.9)	9 (15.8)	23 (9.7)		
Surgical duration, h		3.38 (2.50, 4.58)	3.92 (2.58, 5.00)	3.33 (2.50, 4.57)	−1.432	0.152
Anesthesia duration, h		4.50 (3.50, 5.67)	5.00 (3.58, 6.50)	4.42 (3.50, 5.62)	−1.213	0.225
Frailty score		1.00 (0.00, 2.00)	3.00 (2.00, 3.00)	1.00 (0.00, 2.00)	−8.027	<0.001

Values are presented as *n* (%), mean ± SD, or median (Q1, Q3), as appropriate.

Categorical variables were compared using the chi-square test or Fisher’s exact test.

Continuous variables with approximately normal distribution were compared using the independent-samples t test, whereas non-normally distributed continuous variables were compared using the Mann–Whitney U test.

For nonparametric comparisons, the test statistic shown is the standardized Z value.

### 3.2 Univariate logistic regression analysis for postoperative delirium

Univariate logistic regression analysis showed that age, history of hypertension, history of cerebral infarction, ASA class III, nutritional risk (NRS), intraoperative hypotension, preoperative fasting time, and frailty score were significantly associated with POD (all *p* < 0.05). Higher PNI was associated with a lower risk of POD (OR = 0.909, 95% CI: 0.861–0.959, *p* < 0.001). Regarding educational level, primary school education or below was significantly associated with an increased risk of POD compared with high school/college education or above (OR = 6.093, 95% CI: 2.436–15.240, *p* < 0.001), whereas middle school education was not significantly associated with POD (*p* = 0.171). Serum albumin and lymphocyte count were also significantly associated with POD in univariate analysis (Table 2).

**Table 2:** Univariate logistic regression analysis of factors associated with postoperative delirium.

Predictor	β	SE	Wald	OR	95% CI	*p*
Age (per year increase)	0.169	0.024	49.593	1.184	1.130–1.241	<0.001
Education: middle school (vs high school/college)	−0.709	0.519	1.871	0.492	0.178–1.360	0.171
Education: primary or less (vs high school/college)	1.807	0.468	14.925	6.093	2.436–15.240	<0.001
Hypertension (yes vs. no)	0.858	0.300	8.203	2.360	1.311–4.246	0.004
History of cerebral infarction (yes vs. no)	2.028	0.485	17.508	7.600	2.939–19.652	<0.001
ASA III (vs I–II)	1.830	0.317	33.413	6.231	3.351–11.587	<0.001
Nutritional risk (NRS) (yes vs. no)	1.485	0.311	22.873	4.416	2.402–8.115	<0.001
Intraoperative hypotension (yes vs. no)	1.260	0.323	15.223	3.525	1.872–6.637	<0.001
PNI (per 1-point increase)	−0.096	0.028	12.025	0.909	0.861–0.959	<0.001
Preoperative serum albumin, g/L	−0.105	0.037	8.121	0.901	0.838–0.968	0.004
Lymphocyte count, 10^9^/L	−0.790	0.288	7.553	0.454	0.258–0.797	0.006
Preoperative AST, U/L	0.001	0.001	1.638	1.001	0.999–1.004	0.201
Preoperative fasting time, h	0.803	0.131	37.510	2.231	1.726–2.885	<0.001
C-reactive protein, mg/L	0.009	0.005	3.029	1.009	0.999–1.019	0.082
Frailty score (per 1-point increase)	1.409	0.195	52.277	4.094	2.794–5.998	<0.001

Results are presented as odds ratios (ORs) with 95% confidence intervals (CIs) from univariate logistic regression analyses. OR, odds ratio; CI, confidence interval; ASA, American Society of Anesthesiologists; NRS, Nutritional Risk Screening; PNI, prognostic nutritional index.

### 3.3 Multivariable logistic regression analysis for postoperative delirium

In the multivariable logistic regression model, higher frailty score, older age, history of cerebral infarction, longer preoperative fasting time, and intraoperative hypotension were independently associated with POD. Specifically, the odds of POD increased with frailty score (OR = 2.451, 95% CI: 1.442–4.164, *p* = 0.001), age (OR = 1.108, 95% CI: 1.038–1.182, *p* = 0.002), history of cerebral infarction (OR = 3.774, 95% CI: 1.003–14.195, *p* = 0.049), preoperative fasting time (OR = 2.440, 95% CI: 1.693–3.518, *p* < 0.001), and intraoperative hypotension (OR = 2.868, 95% CI: 1.155–7.122, *p* = 0.023). PNI, primary education or less, hypertension, and ASA III were not statistically significant in the adjusted model. The Hosmer-Lemeshow goodness-of-fit test indicated adequate model calibration (χ^2^ = 6.145, df = 8, *p* = 0.631) (see Table 3).

**Table 3:** Multivariable logistic regression analysis of factors associated with postoperative delirium.

Variable	β	SE	Wald	OR	95% CI	*p*
Frailty score	0.896	0.271	10.980	2.451	1.442–4.164	0.001
PNI (per 1-point increase)	−0.006	0.045	0.017	0.994	0.911–1.085	0.895
Age (per year increase)	0.102	0.033	9.509	1.108	1.038–1.182	0.002
Education: primary or less (1 = yes, 0 = otherwise)	0.528	0.468	1.271	1.695	0.677–4.242	0.260
Hypertension (yes vs. no)	0.172	0.511	0.114	1.188	0.436–3.237	0.736
History of cerebral infarction (yes vs. no)	1.328	0.676	3.861	3.774	1.003–14.195	0.049
ASA III (vs I–II)	0.280	0.512	0.298	1.323	0.485–3.612	0.585
Intraoperative hypotension (yes vs. no)	1.053	0.464	5.151	2.868	1.155–7.122	0.023
Preoperative fasting time, h	0.892	0.187	22.865	2.440	1.693–3.518	<0.001

Multivariable logistic regression analysis was performed to identify factors independently associated with postoperative delirium. Results are presented as β coefficients, standard errors (SE), Wald statistics, odds ratios (ORs), 95% confidence intervals (CIs), and *p* values. Primary education or less was coded as 1 = yes and 0 = otherwise, with high school/college as the reference category. Other reference categories were hypertension (no), history of cerebral infarction (no), ASA physical status (I–II), and intraoperative hypotension (no). Model calibration was adequate according to the Hosmer–Lemeshow goodness-of-fit test (χ^2^ = 6.145, df = 8, *p* = 0.631).

### 3.4 Association between preoperative nutritional status and frailty

To examine the association between preoperative nutritional status and frailty, a multivariable linear regression model was constructed with frailty score as the dependent variable. PNI was significantly negatively associated with frailty score (B = −0.048, 95% CI: −0.067 to −0.029, *p* < 0.001), indicating that lower PNI was associated with more severe frailty. Older age (B = 0.026, 95% CI: 0.015 to 0.036, *p* < 0.001), primary education or less (B = 0.216, 95% CI: 0.006 to 0.426, *p* = 0.044), history of cerebral infarction (B = 0.690, 95% CI: 0.287 to 1.093, *p* = 0.001), and ASA III status (B = 0.341, 95% CI: 0.085 to 0.597, *p* = 0.009) were significantly associated with higher frailty scores. Hypertension and preoperative fasting time were not statistically significant in the final model. No evidence of substantial multicollinearity was observed among the variables included in the model (tolerance: 0.618–0.924; VIF: 1.082–1.619) (see Table 4).

**Table 4:** Multivariable linear regression analysis of factors associated with frailty score.

Variable	B	SE	t	95% CI	*p*
PNI (per 1-point increase)	−0.048	0.010	−4.922	−0.067 to −0.029	<0.001
Age (per year increase)	0.026	0.005	4.823	0.015 to 0.036	<0.001
Education: primary or less (1 = yes, 0 = otherwise)	0.216	0.107	2.022	0.006 to 0.426	0.044
Hypertension (yes vs. no)	−0.014	0.111	−0.127	−0.232 to 0.204	0.899
History of cerebral infarction (yes vs. no)	0.690	0.205	3.373	0.287 to 1.093	0.001
ASA III (vs I–II)	0.341	0.130	2.622	0.085 to 0.597	0.009
Preoperative fasting time, h	0.070	0.040	1.767	−0.008 to 0.148	0.078

Multivariable linear regression analysis was performed to examine the association between preoperative nutritional status and frailty score. B represents the unstandardized regression coefficient. Primary education or less was dummy coded as 1 = yes, 0 = otherwise. No evidence of substantial multicollinearity was observed among the variables included in the model, with tolerance values ranging from 0.618 to 0.924 and VIF values ranging from 1.082 to 1.619. PNI, prognostic nutritional index; ASA, American Society of Anesthesiologists; VIF, variance inflation factor.

### 3.5 Mediation analysis

Since PNI was significantly associated with frailty score in the adjusted linear regression model, mediation analysis was further performed using PROCESS macro version 5.0 (Model 4) to examine whether frailty showed a significant indirect effect in the association between PNI and POD. After adjustment for age, hypertension, history of cerebral infarction, primary education or less, ASA III status, and preoperative fasting time, PNI remained significantly associated with frailty score (B = −0.0476, SE = 0.0097, *p* < 0.001). In the logistic outcome model, frailty score was significantly associated with POD (B = 0.9032, SE = 0.2614, *p* = 0.0005), whereas the direct effect of PNI on POD was not statistically significant (B = −0.0039, SE = 0.0439, *p* = 0.9295). There was significant indirect effect of PNI on POD through frailty (effect = −0.0430, BootSE = 0.0177, 95% bootstrap CI: −0.0866 to −0.0187), indicating an indirect-only mediation pattern (Table 5). As a sensitivity analysis, preoperative C-reactive protein was additionally included in the mediation model. The indirect effect of PNI on POD through frailty remained statistically significant (effect = −0.0347, 95% bootstrap CI: −0.0735 to −0.0132), indicating that the indirect effect remained statistically significant after additional adjustment for CRP.

**Table 5:** Mediation analysis of frailty in the association between prognostic nutritional index and postoperative delirium.

Effect	Estimate	Standard error	95% bootstrap CI	*p*
Direct effect (PNI → POD)	−0.0039	0.0439	−0.0900 to 0.0822	0.9295
Indirect effect (PNI → frailty → POD)	−0.0430	0.0177	−0.0866 to −0.0187	-

Mediation analysis was performed using PROCESS macro version 5.0 (Model 4) with 5,000 bootstrap resamples. The model included age, hypertension, history of cerebral infarction, primary education or less, ASA III status, and preoperative fasting time as covariates. Because postoperative delirium (POD) was a binary outcome, effects on Y are expressed on a log-odds scale. The standard error reported for the indirect effect is the bootstrap standard error. Statistical significance of the indirect effect was determined by whether the 95% bootstrap confidence interval excluded zero; therefore, no separate *p* value is reported for the indirect effect.

### 3.6 ROC analysis of PNI, frailty score, and their combined model for predicting postoperative delirium

**Figure 1:**
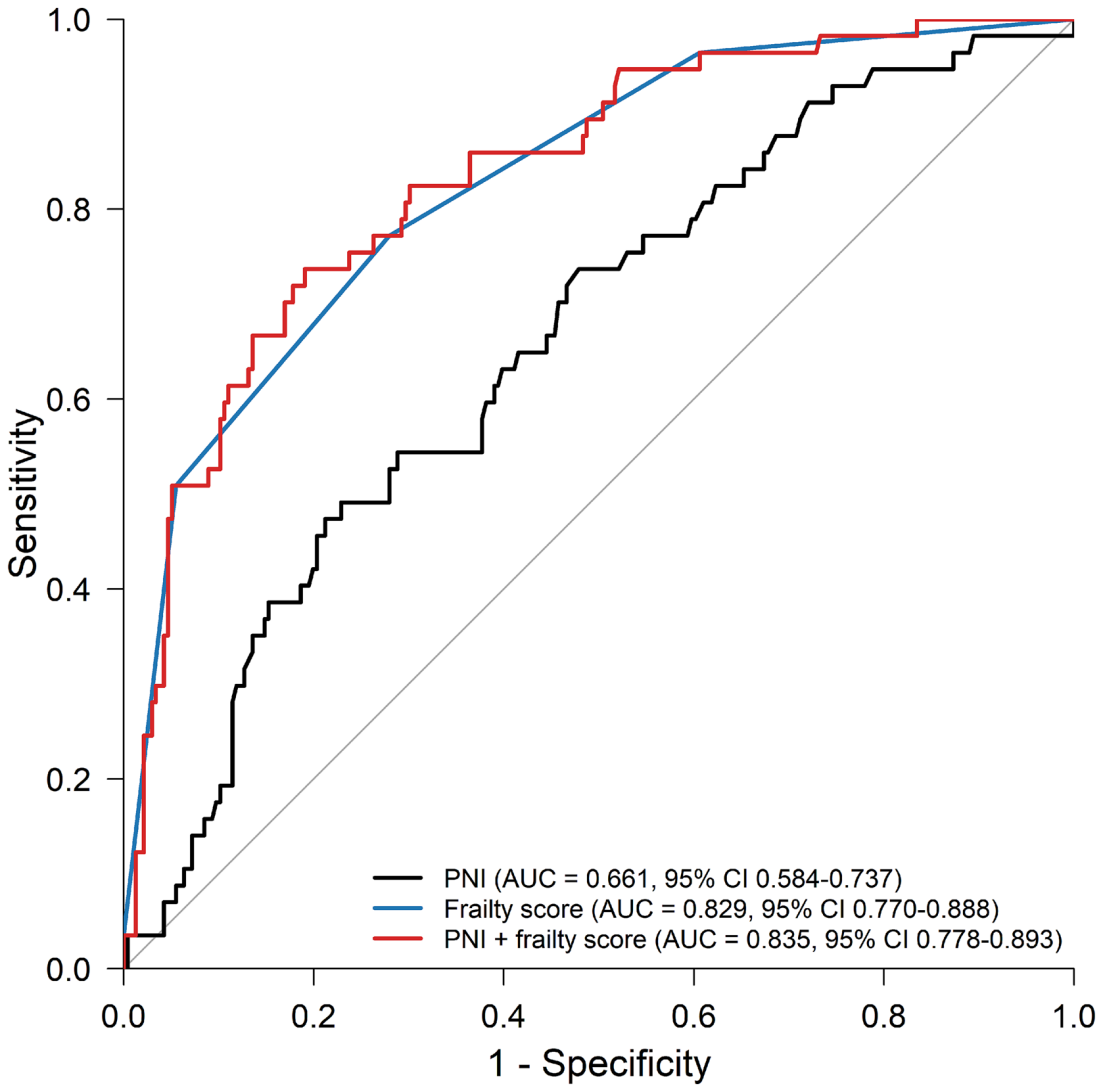
Receiver operating characteristic (ROC) curves of PNI, frailty score, and the combined model for predicting postoperative delirium.

ROC curve analysis was performed to evaluate the discriminative performance of PNI, frailty score, and their combined model for POD. The AUC of PNI was 0.661 (95% CI: 0.584–0.737, *p* < 0.001), with a sensitivity of 0.491 and a specificity of 0.771 at the optimal cut-off value of 43.55. The frailty score showed better predictive performance, with an AUC of 0.829 (95% CI: 0.770–0.888, *p* < 0.001), a sensitivity of 0.772, and a specificity of 0.720 at the optimal cut-off value of 2. When PNI and frailty score were combined, the AUC increased slightly to 0.835 (95% CI: 0.778–0.893, *p* < 0.001), with a sensitivity of 0.737 and a specificity of 0.809 at the optimal cut-off value of 0.29. According to the DeLong test, the AUC of frailty score was significantly higher than that of PNI (Z = 4.067, *p* < 0.001), whereas the combined model did not significantly improve discrimination compared with frailty score alone (Z = 0.717, *p* = 0.473) (see Figure 1, Table 6).

**Table 6:** Receiver operating characteristic (ROC) analysis of PNI, frailty score, and their combined model for predicting postoperative delirium.

Model	AUC	95% CI	Sensitivity	Specificity	Cut-off	*p* value
PNI	0.661	0.584–0.737	0.491	0.771	43.55	<0.001
Frailty score	0.829	0.770–0.888	0.772	0.720	2	<0.001
PNI + Frailty score	0.835	0.778–0.893	0.737	0.809	0.29	<0.001

ROC curve analysis was performed to evaluate the predictive performance of PNI, frailty score, and their combined model for postoperative delirium. The combined model score was derived from the predicted probability of a logistic regression model including PNI and frailty score. The optimal cut-off value was determined using the Youden index. For ROC analysis of PNI, the direction was reversed. Thus, lower PNI values indicated higher POD risk; the reported cut-off was converted back to the original PNI scale. *p* values test whether the AUC differs from 0.5. Pairwise comparisons of AUCs were performed using the DeLong test. The AUC of frailty score was significantly higher than that of PNI (Z = 4.067, *p* < 0.001), whereas the combined model did not significantly improve discrimination compared with frailty score alone (Z = 0.717, *p* = 0.473).

The combined model was derived from the predicted probability of a logistic regression model including PNI and frailty score. The AUCs were 0.661 for PNI, 0.829 for frailty score, and 0.835 for the combined model. According to the DeLong test, the AUC of frailty score was significantly higher than that of PNI (*p* < 0.001), whereas the combined model did not significantly improve discrimination compared with frailty score alone (*p* = 0.473).

## 4 Discussion

### 4.1 Principal findings

This study explored the associations among preoperative nutritional status, frailty, and postoperative delirium (POD) in patients undergoing pancreatic cancer surgery. Frailty was independently associated with POD, and poorer preoperative nutritional status, as reflected by a lower prognostic nutritional index (PNI), was significantly associated with greater frailty. Mediation analysis suggested that frailty may help explain the association between poorer nutritional status and POD. Although combining PNI with frailty yielded better discrimination than PNI alone, it did not confer a statistically significant incremental benefit over frailty alone. Previous studies mainly focus on baseline comorbidities and perioperative factors related to POD. However, the interrelationship among nutritional status, frailty, and POD has been less well studied (6, 13). The findings suggest that poorer nutritional status may be associated with a higher risk of POD, and this association may be partly accounted for by frailty.

### 4.2 Frailty and postoperative delirium

The present study identified frailty as an independent factor associated with POD in patients undergoing pancreatic cancer surgery (OR = 2.451, 95% CI: 1.442–4.164, *p* = 0.001). This finding is consistent with previous evidence showing that frailty is strongly associated with adverse postoperative outcomes, including increased complications, prolonged hospital stay, and higher mortality (25). In surgical populations, a growing body of evidence further indicates that frail patients have a significantly higher risk of POD than non-frail patients. A previous meta-analysis reported that preoperative frailty was associated with significantly greater odds of POD in older elective surgical patients, supporting the role of frailty as an important marker of perioperative vulnerability (13–15). Frailty reflects a state of reduced physiological reserve and multisystem functional decline. Patients with frailty often present with chronic low-grade inflammation, impaired immune function, and metabolic dysregulation, which may increase susceptibility to acute neurocognitive complications under surgical stress. These physiological alterations may exacerbate neuroinflammatory responses and impair blood–brain barrier function, thereby increasing vulnerability to acute brain dysfunction. Therefore, frailty may be viewed as an important clinical indicator of overall physiological vulnerability in relation to perioperative neuropsychiatric complications.

### 4.3 Nutritional status and frailty

Malnutrition is common in patients with pancreatic cancer and plays an important role in the development of frailty. It has been reported that patients with pancreatic cancer frequently experience substantial changes in body composition, including increased fat mass and/or reduced skeletal muscle mass. A higher fat-to-lean mass ratio has been associated with poorer long-term survival, suggesting that nutritional status and body composition are important determinants of prognosis (16). In addition, pancreatic tumors and pancreatic dysfunction may impair digestion and nutrient absorption and contribute to metabolic disturbances and systemic inflammation. This further increases the risk of protein–energy malnutrition and muscle loss (17). The prognostic nutritional index (PNI), calculated from serum albumin levels and peripheral lymphocyte counts, has been widely used as a convenient indicator of immunonutritional status. Indeed, low PNI levels are associated with increased postoperative complications and poorer survival outcomes in patients with various cancers (23, 29, 30). Malnutrition is regarded as one of the biological foundations of frailty. Inadequate nutritional intake may lead to muscle loss, impaired immune function, and chronic low-grade inflammation, thereby accelerating the progression of frailty. Consistent with these findings, the present study showed that PNI was significantly associated with frailty score (B = −0.048, 95% CI: −0.067 to −0.029, *p* < 0.001), indicating that poorer nutritional status was associated with more severe frailty.

### 4.4 Potential mechanisms linking nutrition, frailty, and delirium

In this work, PNI was not independently associated with POD after adjustment. Nevertheless, previous systematic reviews and meta-analyses have reported that lower preoperative PNI levels are generally associated with an increased risk of POD (31). Combined with our mediation findings, this may indicate that the association between poorer nutritional status and POD is expressed mainly through frailty rather than through a stable independent direct effect of PNI on POD. Methodological studies have shown that a significant indirect effect may be observed even when the total effect or direct effect is not significant; when the indirect effect exists in the absence of a direct effect, this pattern can be classified as indirect-only mediation (32, 33).

From a biological perspective, malnutrition may promote frailty through skeletal muscle loss, impaired protein synthesis, immune dysfunction, and a persistent low-level inflammatory state. Because of reduced physiological reserve and impaired adaptability to surgical stress, frail patients may be more vulnerable to inflammatory, metabolic, and neurocognitive disturbances after surgery. In addition, the gut–brain axis has been proposed as a potential mechanism linking nutritional status, frailty, and brain function (18). Through interactions among the gut microbiota, intestinal barrier, immune system, and central nervous system, nutritional impairment may contribute to gut microbiota dysbiosis, barrier dysfunction, and systemic inflammation (19, 20). Previous studies have also suggested that intestinal dysfunction and gut microbiota imbalance may promote frailty progression through impaired nutrient absorption and chronic inflammation (21), while the concept of “gut frailty” further supports the idea that gut dysbiosis and intestinal dysfunction may influence both muscle metabolism and cognitive function (22). Therefore, poorer nutritional status may be associated with greater susceptibility to POD, possibly in relation to frailty-related reductions in physiological resilience. However, because this was a prospective observational study, these findings should be interpreted as supporting a plausible intermediate pathway rather than definitive evidence of causality (33).

### 4.5 Predictive value of nutritional status and frailty

This study further evaluated the discriminative ability of nutritional status and frailty for POD, whereas frailty score showed better discriminative performance. Although combining PNI with frailty slightly increased the AUC compared with frailty alone, this increment was not statistically significant. These findings are generally consistent with previous systematic reviews and meta-analyses. Hung et al. (31) reported that lower preoperative PNI levels were associated with an increased risk of POD, suggesting that PNI may serve as a convenient marker in perioperative risk assessment. Combined with the current study’s findings, PNI alone may not fully reflect the overall physiological reserve of patients, and its incremental predictive value beyond frailty appears limited. Therefore, nutritional assessment may be more appropriately viewed as a complement to frailty screening, rather than as a factor that meaningfully improves POD risk prediction over frailty assessment alone.

### 4.6 Clinical implications and limitations

From a clinical perspective, PNI is derived from serum albumin levels and peripheral lymphocyte counts and can be readily obtained from routine laboratory tests, making it a practical indicator in perioperative assessment. Previous meta-analyses have suggested that lower preoperative PNI is associated with an increased risk of POD, supporting its potential role as an easily available screening marker. However, although PNI showed moderate discriminative ability, its incremental predictive value beyond frailty was limited. Therefore, in patients with low PNI, frailty assessment and attention to nutritional status may be considered during perioperative evaluation. In summary, nutritional assessment may be more appropriately viewed as a complement to frailty screening, rather than as a substitute, for identifying patients at increased risk of POD after pancreatic cancer surgery.

Several limitations should be acknowledged. First, this was a single-center prospective observational study with a relatively modest sample size, which may limit the generalizability and stability of the findings. Second, although several clinically relevant covariates were adjusted for, residual confounding cannot be excluded, particularly because baseline cognitive status, some intraoperative variables, and ICU-related factors were not systematically incorporated into the analyses. Third, although PNI and frailty were assessed preoperatively and POD was evaluated postoperatively, the mediation results should be interpreted cautiously and should not be considered definitive causal evidence. Finally, frailty was assessed using the FRAIL scale. This is practical for clinical screening but may not capture frailty as comprehensively as more detailed instruments. Future multicenter studies with larger samples and more comprehensive perioperative assessment are needed to validate these findings.

## Data Availability

The original contributions presented in the study are included in the article/supplementary material, further inquiries can be directed to the corresponding authors.
